# Evaluating Primers for Profiling Anaerobic Ammonia Oxidizing Bacteria within Freshwater Environments

**DOI:** 10.1371/journal.pone.0057242

**Published:** 2013-03-07

**Authors:** Puntipar Sonthiphand, Josh D. Neufeld

**Affiliations:** Department of Biology, University of Waterloo, Waterloo, Ontario, Canada; Auburn University, United States of America

## Abstract

Anaerobic ammonia oxidizing (anammox) bacteria play an important role in transforming ammonium to nitrogen gas and contribute to fixed nitrogen losses in freshwater environments. Understanding the diversity and abundance of anammox bacteria requires reliable molecular tools, and these are not yet well established for these important Planctomycetes. To help validate PCR primers for the detection of anammox bacteria within freshwater ecosystems, we analyzed representative positive controls and selected samples from Grand River and groundwater sites, both from Ontario, Canada. The objectives of this study were to identify a suitable anammox denaturing gradient gel electrophoresis (DGGE) fingerprint method by using GC-clamp modifications to existing primers, and to verify the specificity of anammox-specific primers used for DGGE, cloning and qPCR methods. Six primer combinations were tested from four published primer sets (i.e. A438f/A684r, Amx368f/Amx820r, An7f/An1388r, and Pla46/1392r) for both direct and nested PCR amplifications. All PCR products were run subsequently on DGGE gels to compare the resulting patterns. Two anammox-specific primer combinations were also used to generate clone libraries and quantify anammox bacterial 16S rRNA genes with qPCR. The primer set A438f/A684r was highly specific to anammox bacteria, provided reliable DGGE fingerprints and generated a high proportion of anammox-related clones. A second primer set (Amx368f/Amx820r) was anammox specific, based on clone library analysis, but PCR products from different candidate species of anammox bacteria resolved poorly using DGGE analysis. Both DGGE and cloning results revealed that *Ca.* Brocadia and an uncharacterized anammox bacterial cluster represented the majority of anammox bacteria found in Grand River sediment and groundwater samples, respectively. Together, our results demonstrate that although Amx368f/Amx820r was useful for anammox-specific qPCR and clone library analysis, A438f/A684r was the most suitable primer set for multiple molecular assessments of anammox bacteria in freshwater environments.

## Introduction

Even before the discovery of anaerobic ammonia oxidizing (anammox) bacteria as Planctomycetes [1] and elucidation of their metabolic pathways, physiology and morphology [2]–[3], the anammox process was recognized by nutrient profiles and thermodynamic calculations [4]–[6]. Anammox bacteria have the ability to transform ammonium into nitrogen gas by using nitrite as an electron acceptor under anoxic conditions. Anammox bacteria were first discovered in a laboratory-scale reactor in 1995 [7]. Since then, many reports have demonstrated the widespread occurrence and contribution of anammox bacteria in a variety of natural ecosystems [8]–[12]. Anammox bacteria are important in the global nitrogen cycle, and responsible for high nitrogen losses: ∼50% in marine sediments [13]–[16], ∼40% in contaminated groundwater [17] and 4–37% in terrestrial habitats [18]. These findings demonstrate the important role of anammox bacteria in natural environments.

There are still no pure culture isolates of anammox bacteria due to their extremely slow growth rates, relatively low biomass yields and inactivation by low concentrations of oxygen and nitrite [19]–[21]. Culture-independent methods such as 16S rRNA gene-based analysis [22]–[24] and fluorescence *in situ* hybridization (FISH) [2], [25]–[26] are the common methods used for anammox bacterial community analysis. The 16S rRNA genes of known anammox bacteria show several phylogenetically distinct *Candidatus* genera including *Ca.* Brocadia, *Ca.* Scalindua, *Ca.* Kuenenia, *Ca.* Anammoxyglobus and *Ca.* Jettenia. The average sequence similarity between *Ca.* Scalindua and *Ca.* Brocadia or *Ca.* Kuenenia clusters is only 85% [3], [26]. Thus, it is very challenging to design primer sets that target all known anammox genera. Although several anammox-specific primers have been used for 16S rRNA gene amplification, reported problems include low recovery efficiencies of anammox-related clones, non-specific amplification and an inability to target all anammox bacterial clusters [8], [22], [24], [27]–[32]. Anammox bacteria from groundwater have recently been profiled by comparing bacteria-specific denaturing gradient gel electrophoresis (DGGE) patterns (341f-GC/518r) with “anammox specific” patterns from a nested PCR protocol (An7f/An1388r followed by 341f-GC/518r; [17]). The intense bands that appeared in DGGE fingerprints were confirmed to be related to anammox bacteria.

Since identifying the contribution of anammox bacteria to fixed nitrogen losses in natural ecosystems [33], much research has focused on studying anammox bacteria in marine environments [34]. Since anammox bacteria were first reported in freshwater environments [35], there have been only five known studies characterizing anammox bacterial communities in freshwater habitats [17], [22], [29], [35]–[36]. Thus, information on the diversity, abundance and activity of anammox bacteria in freshwater ecosystems is still scarce. In this study, we focused on PCR primer-based detection methods for anammox bacteria within freshwater environments, including samples taken from the Grand River and from a previously studied groundwater site.

The primer set, A438f and A684r, successfully quantified anammox bacteria 16S rRNA gene copies in wetland soils [37] but was not tested for DGGE prior to this study. Another primer set, Amx368f and Amx820r, was commonly used to detect anammox bacteria in various environments. Both primers were originally designed for FISH probes and were then applied as forward and reverse PCR primers for detecting anammox bacteria by clone library analysis in freshwater, terrestrial and marine ecosystems, such as Lake Kitaura [36], groundwater [17], fertilized paddy soil [18], constructed wetland [38], peat soil [39], Cape Fear River estuary [23], coastal marine sediment [27], the Jiaojiang estuary [40], the South China Sea [41], and a high-temperature petroleum reservoir [42]. In this study, these existing PCR primers were modified with GC clamps and tested for the ability to generate anammox bacterial 16S rRNA gene DGGE fingerprints. The other two primer sets investigated here (An7f/An1388r and Pla46/1392r) amplified a large amplicon (∼1,400 bp). The primers An7f and An1388r were designed originally to target anammox bacteria in freshwater and marine sediments [22]. The Planctomycetes-specific FISH probe, Pla46, has been used as a forward primer with the reverse universal primer 1392R to obtain PCR products that were subsequently used for nested PCR templates in both natural [12], [28], [30], [43] and artificial [44]– environments.

The main objectives of this study were (i) to identify suitable PCR primer combinations for DGGE assessment of anammox bacterial communities and (ii) to compare the efficiency and specificity of the existing primer sets for DGGE, clone library and qPCR assays. The results provide important experimental validation for using specific primer sets for investigating the diversity and abundance of anammox bacteria within freshwater environments.

## Methods

### Sampling site description and sample collection

The Grand River is located in southwestern Ontario, Canada. This large river and its tributaries receive high nitrogen inputs, mainly from agricultural runoff and wastewater discharge. Two sampling sites along the Grand River (Bridgeport and Blair), located in the city of Waterloo, were chosen as representative freshwater environmental sites. Sediment (SedBr and SedBl), epilithic biofilm (EpBr and EpBl) and water (WaBr and WaBl) samples were collected from each sampling site in June 2010. In addition, groundwater samples were collected from the Zorra township, Ontario (site details and sampling information were previously described in [17]). Both groundwater (GW) and groundwater sediment core (GS) samples were collected at a 7.5-m depth in July 2009. All water samples of approximately 300 ml were filtered onsite onto 0.22-µm Sterivex filters (Millipore, USA). All samples were stored on dry ice during transportation and kept at −80°C until DNA extraction. The environmental chemistry analyses from each site were shown in Table 1.

**Table 1:** Water chemistry for each sample site.

Sampling site	NH_4_^+^N	NO_2_^−^N	NO_3_^−^N	DO	pH
	(mg NH_4_^+^ L^−1^)	(mg NO_2_^−^ L^−1^)	(mg NO_3_^−^ L^−1^)	(mg L^−1^)	
Bridgeport	0.05	0.04	2.06	7.9	8.14
Blair	0.47	0.46	1.80	7.3	7.91
Zorra*	ND	NA	10	1.97	7.04

NA = Not available; ND = Not detected;

* Samples from Zorra site were collected in July, 2010 but all parameters reported were measured in August 2010.

### DNA extraction

Genomic DNA was extracted from Grand River sediment and epilithic biofilm samples using the MoBio PowerSoil DNA kit (MoBio Laboratories, USA), following the manufacturer's protocol. Nucleic acids from all Sterivex filters were extracted according to a previously published protocol [46]. The quality and quantity of extracted DNA were measured by agarose gel electrophoresis and spectrophotometry (NanoDrop Spectrophotometer ND-100; Thermo Fisher Scientific, USA), respectively. Extracted DNA was then diluted to 5–10 ng µl^−1^ for use as PCR template.

### Denaturing gradient gel electrophoresis (DGGE)

All samples were PCR amplified with bacteria-specific primers (341f-GC/518r), targeting the bacterial 16S rRNA gene. This PCR, in addition to a nested PCR approach for detecting anammox bacteria, followed previously published protocols [17]. Briefly, for the nested PCR, template was amplified with anammox bacteria-specific primers An7f/An1388r, followed by amplification by the bacteria-specific DGGE primers 341f-GC/518r. Additional published primers were also modified with GC-clamps for DGGE assessment [47]. These anammox-specific nested PCR amplifications involved either An7f/An1388r or Pla46/1392r for the first round of PCR, followed by anammox-specific A438f-GC/A684r or Amx368f-GC/Amx820r for a second reaction. The PCR components contained 2.5 µl of 10× ThermoPol Reaction Buffer, 0.05 µl of forward and reverse primer (100 µM stocks), 0.05 µl of dNTPs (100 mM stock), 0.1 µl of *Taq* DNA polymerase (5 U µl^−1^ stock), 1.5 µl of bovine serum albumin (10 mg ml^−1^ stock) and 1 µl of DNA template (representing 5–10 ng of genomic DNA) in a total reaction volume of 25 µl. All PCR amplifications were carried out with an initial denaturation at 95°C for 5 min, followed by primer-set-specific thermal cycling conditions (Table 2) with a total of 30–35 cycles for the first round PCR and a final extension of 72°C for 10 min to complete the reaction. The first round PCR products were diluted 100-fold to serve as template for the nested PCR. Nested PCR conditions and thermal cycle profiles of each primer set were the same as previously described, except for the number of PCR cycles. All nested PCR were run for a total of 20–25 cycles. After each amplification, PCR products were verified by agarose gel electrophoresis to confirm amplicon size. The 341f-GC/518r and A438f-GC/A684r PCR products were run on 10% acrylamide gels, with 30%-70% denaturing gradients. The Amx368f-GC/Amx820r PCR products were profiled on 8% acrylamide gels, with 30%-70% denaturing gradients. All DGGE gels were run for 15 h at 85 V and at 60°C, using a DGGEK-2401 (CBS Scientific Company, USA). The DGGE gels were stained with SYBR green (Invitrogen, USA) and scanned with a Pharos FXTM Plus Molecular Imager (Bio-Rad, USA). Representative bands were excised and sequenced by the corresponding anammox-specific primers at Beckman Coulter Genomics using an ABI 3730XL sequencer.

**Table 2:** Summary of the PCR primers and conditions used in this study.

Primer^1^	Specificity	*E. coli* position	PCR conditions			Reference
			Denaturation	Annealing	Extension	
A438f^2^	All anammox bacteria	438–455	95°C, 30 sec	55°C, 30 sec	72°C, 30 sec	[37]
A684r^2^		667–684				
Amx368f	All anammox bacteria	368–385	95°C, 45 sec	59°C, 45 sec	72°C, 45 sec	[26]
Amx820r	*Ca.* Kuenenia, *Ca.* Brocadia	820–841				[25]
An7fAn1388r	*Ca.* Kuenenia, *Ca.* Brocadia, *Ca.* Scalindua	7–261372–1388	95°C, 45 sec	63°C, 1 min	72°C, 1 min	[22]
Pla46	Planctomycetes	46–63	95°C, 45 sec	59°C, 1 min	72°C, 1 min	[62]
1392r	Universal bacteria	1392–1406				[63]
341f	Universal bacteria	341–357	95°C, 30 sec	55°C, 30 sec	72°C, 30 sec	[64]
518r		518–534				

1 For DGGE, a GC-clamp was attached to the forward primers for PCR (A348f-GC, Amx368f-GC and 341f-GC).

2 The qPCR conditions were exactly the same (primer concentrations, annealing temperature and without additional BSA) as the original condition [37].

### Clone library analysis

Three representative samples (SedBr, SedBl and GW) were selected to generate clone libraries with three anammox primer sets (An7f/An1388r, A438f/A684r and Amx368f/Amx820r) to compare the efficiency and specificity of each primer set. The PCR conditions for cloning were the same as those described for DGGE (Table 2). The PCR products were ligated and transformed using a TOPO TA Cloning kit and One Shot TOP10 Chemically Competent cells (Invitrogen, USA), respectively, according to manufacturer's protocols. Between 30–70 white colonies were selected from each library and screened for the presence of inserts by each anammox-specific PCR primer set prior to being sequenced at Beckman Coulter Genomics, USA as previously mentioned in the DGGE section.

### Quantitative real-time PCR (qPCR)

Anammox bacterial 16S rRNA genes were quantified by two specific primer sets (A438f/A684r and Amx368f/Amx820r) for comparison. Total bacterial abundance was also investigated by primers 341f/518r as a reference. The qPCR master mix contained 5 µl of SsoAdvanced SYBR Green Supermix (Bio-Rad, USA), 0.03 µl of each primer (100 µM stocks), 0.02 µl of bovine serum albumin (10 mg ml^−1^ stock) and 1 µl of genomic DNA template (5–10 ng stock) in a total volume of 10 µl. All qPCR amplifications were performed in duplicate on a CFX96 real-time system (Bio-Rad, USA). Although specific annealing temperatures were used (Table 2), qPCR thermal programs were common for all three primer sets. An initial denaturation at 98°C for 2 min was followed by 35 cycles of 98°C for 5 sec, annealing at the primer-specific annealing temperature for 30 sec and 72°C for 30 sec, with a plate read after each cycle. Following PCR, melt curves were generated between 65°C–95°C in 0.5°C increments to ensure PCR specificity. Reference freshwater samples with high anammox abundance were amplified by each specific primer set, pooled by primer set, then purified to serve as anammox bacterial standard templates for qPCR. For general bacterial qPCR, the standard curves were constructed from *Escherichia coli* genomic DNA. Each PCR product was purified using a MinElute kit (Qiagen, USA) and quantified by the NanoDrop Spectrophotometer ND-100. Ten-fold serial dilutions were applied to the standard DNA PCR product template to create the qPCR standard curves, which was linear between 10^1^–10^7^ copies, with efficiencies of 84–93% and coefficients of determination (*R* ^2^)≥0.996 for all standard curves. The specificity of qPCR amplification was confirmed by melting curve analysis and agarose gel electrophoresis of all products after each run.

### Statistical and phylogenetic analysis

All analyzed sequences from clone libraries were clustered into operational taxonomic units (OTUs), based on 1% and 3% dissimilarity cut-off settings for 16S rRNA gene nucleotide similarity by AXIOME version 1.5.0 [48]. All clone sequences showing 97% and 99% identical sequences were grouped together before constructing two phylogenetic trees, based on differing distance levels in nucleotide sequences, to compare anammox bacterial clusters and tree topologies. All sequences from DGGE and clone library analysis were compared to the Genbank non-redundant database with the Basic Local Alignment Search Tool (BLAST) to identify related sequences. All DGGE bands and representative clones from each library were aligned with selected uncultured anammox bacteria sequences and reference *Ca.* anammox bacteria species using MUSCLE [49]. A phylogenetic tree was constructed in PhyML v.3.0.1 [50]–[51], with the GTR model. The approximate likelihood ratio test (aLRT) statistic was used to calculate branch support values. Phylogenetic trees were run with five random starts to optimise the tree topology.

### Nucleotide accession number

All anammox bacterial 16S rRNA gene sequences were deposited in GenBank with accession numbers JX392915-JX392948.

## Results

### Anammox bacterial primers for DGGE

**Figure 1:**
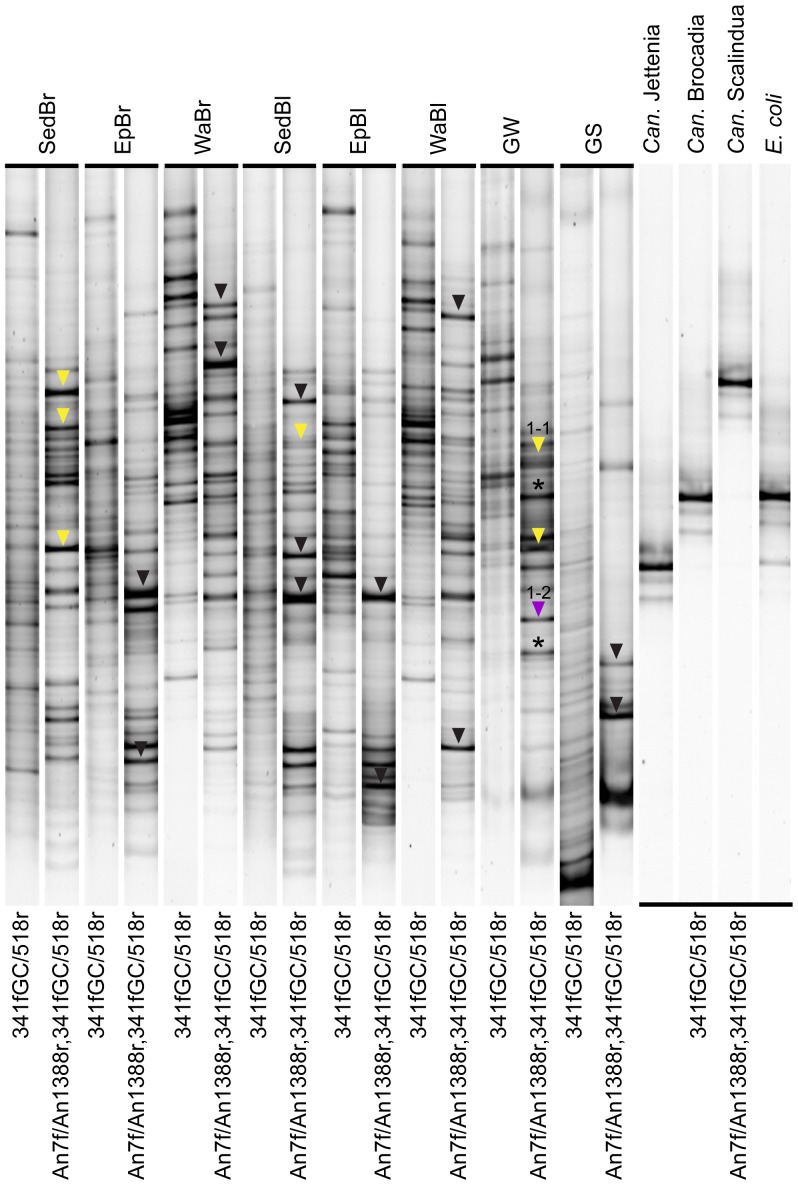
Denaturing gradient gel electrophoresis (DGGE) profiles of bacterial and anammox bacterial 16S rRNA genes in comparison. Together with six environmental samples, positive control template (*Ca.* Jettenia, *Ca.* Brocadia and *Ca.* Scalindua) and a negative control (*E. coli*) were included. Triangles indicate sequenced bands. Band 1-1, indicated by a yellow triangle, was associated with Unknown anammox cluster 1 [17] and band 1-2, indicated by a purple triangle, was affiliated with Unknown anammox cluster 2 [17]. Bands indicated by black triangles were not affiliated with anammox bacteria. A star indicates a band with low quality sequence, which was excluded from subsequent phylogenetic analysis.

**Figure 2:**
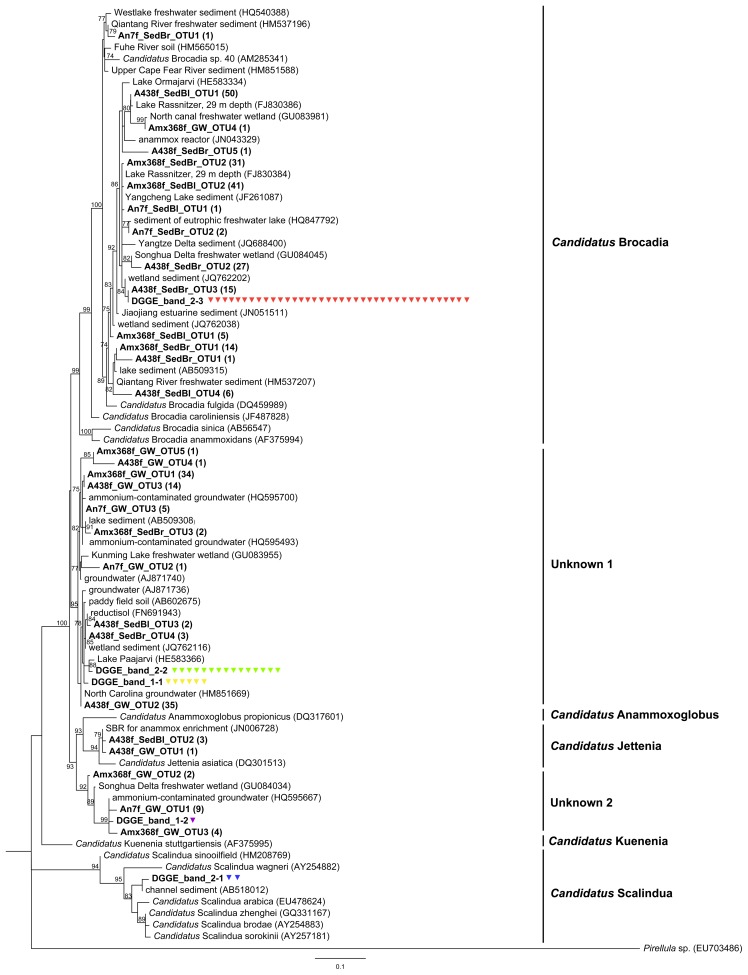
Phylogenetic tree of anammox bacterial 16S rRNA genes retrieved from both DGGE and clone library methods (shown in bold). All DGGE band sequences are indicated by “DGGE” with representative band ID, related to Fig. 1 and 3. The number of individual DGGE bands is indicated by the triangles with corresponding colour. Clone sequences with 97% identity from each library were grouped together; the representative clones from each operational taxonomic unit (OTU) were included in the analysis. The number of sequences belonging to each OTU is indicated in parentheses. The maximum likelihood tree was constructed with the GTR model. Branch support values (aLRT) greater than 50% are indicated at the nodes.

In a previous study, DGGE, qPCR and Illumina 16S rRNA gene data indicated that anammox bacteria were numerically important community members of an ammonium-contaminated groundwater site (Zorra, Ontario). Our initial benchmarking experiment for this study was to repeat the DGGE protocol with DNA extracts from this groundwater at a 7.5-m depth, and include additional sediment, epilithic biofilm and water samples from two representative sites within the Grand River, which are not as strongly dominated by anammox bacteria [52]. As positive controls, we used plasmids carrying anammox 16S rRNA genes associated with *Ca.* Jettenia, *Ca.* Brocadia and *Ca.* Scalindua; genomic DNA from *E. coli* was used as a negative control. The results demonstrated that in all cases, patterns generated by bacteria-specific primers (341f-GC/518r) were distinct from those generated by the nested anammox PCR protocol for DGGE (Fig. 1). Of the 20 bands selected for sequencing, all three analyzed bands from SedBr, one band from SedBl and three bands from GW were affiliated with anammox bacteria (Fig. 1), demonstrating that this nested PCR design resulted in the enrichment of anammox bacterial amplicons. Genbank BLAST analysis results showed that the sequences were 97%–100% identical to previously reported sequences recovered from sediment in wetlands (JQ762203) and ammonium-contaminated groundwater from the same site analyzed in this study (HQ595700 and HQ595667). Phylogenetic analysis revealed that all six bands indicated by a yellow triangle were associated with Unknown 1 cluster, while another band indicated by a purple triangle fell into Unknown 2 cluster (Fig. 1 and Fig. 2). Importantly, non-specific amplification of the *E. coli* 16S rRNA gene was observed (Fig. 1), which was a non-specific amplification problem also seen in most of the freshwater samples included in this study. Bands indicated by black triangles were not related to anammox bacteria. These bands were 97%–100% identical to Actinobacteria, Chloroflexi and Cyanobacteria. These results demonstrate that a coupling of an anammox-specific amplification using An7f/An1388r with bacteria-specific PCR for DGGE (341f-GC/518r) was not suitable for targeting anammox bacteria in the environmental samples at the expense of all other bacteria, especially if anammox bacterial abundances were relatively low, such as in the Grand River epilithic biofilm and water column samples. *In silico* analysis (Table S1) revealed that An7f showed two mismatches at the 5′end and one, none and three mismatches at 3′end against Actinobacteria, Chloroflexi, and *E. coli* 16S rRNA gene sequences, respectively. Although, An1388r showed many mismatches for Actinobacteria, Chloroflexi, and *E. coli*, it was highly specific for both anammox and non-anammox of Planctomycetales 16 rRNA gene sequences. This analysis supported that An7f/An1388r could amplify non-anammox bacterial sequences. Note that band positions on a DGGE gel were different for the three anammox *Ca.* genera (Fig. 1), which is useful for distinguishing different anammox populations if non-specific amplification is not a concern.

**Figure 3:**
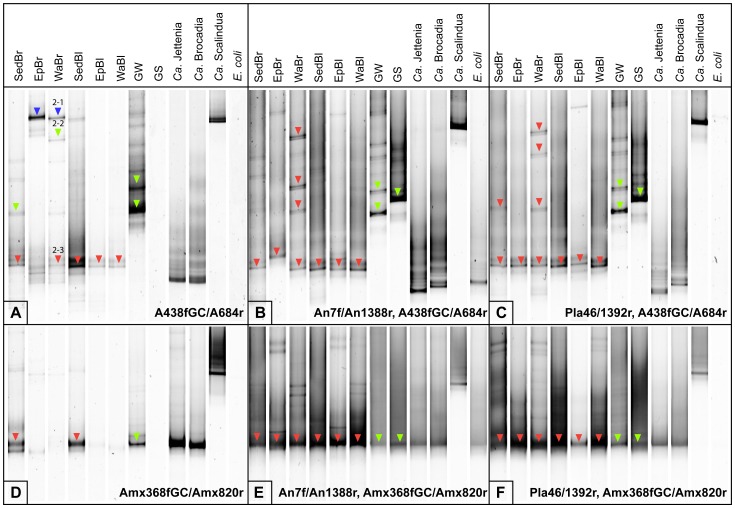
Denaturing gradient gel electrophoresis (DGGE) profiles of anammox bacterial 16S rRNA genes. The two main primer sets were used to generate the anammox-fingerprints (A and D) by direct PCR amplification. The additional four patterns (B, C, E and F) were produced by the nested PCR assay, using other two primer sets and followed by the two main sets. Triangles indicate a total of 55 bands, associated with anammox bacteria. Only three representative bands were shown in the phylogenetic tree. Bands 2-1, 2-2 and 2-3 were indicated by the blue, green and red triangles, respectively. Each coloured triangle indicates exactly the same phylogeny shown in Fig. 2.

Because many bands from the previously published nested DGGE approach were not affiliated with anammox bacteria in the Grand River epilithic biofilm and water samples (Fig. 1), we combined alternative published primer sets to generate DGGE profiles from the selected freshwater environments. The two tested primer sets were A438f-GC/A684r and Amx368f-GC/Amx820r; neither primer set had been tested with GC clamps for DGGE prior to this study. A nested PCR technique was also included in this comparison to identify whether this approach, useful for samples with low target abundance, could increase the sensitivity of anammox bacterial 16S rRNA gene signals without altering the profiles generated. We used primers An7f/An1388r, which targets a near full-length (∼1,400 bp) region of the anammox bacterial 16S rRNA gene associated with *Ca.* Scalindua, *Ca.* Brocadia and *Ca.* Kuenenia genera [22]. Primers Pla46/1392r, targeting all bacterial 16S rRNA genes within the Planctomycetes phylum (∼1,400 bp), were also used for the first PCR amplification for comparison. In all nested PCR assays, after generating a larger amplicon from the initial PCR, a shorter fragment was amplified by more specific anammox bacterial primers with the GC-clamp. All freshwater samples and both positive and negative controls were amplified by direct PCR using the two main primer sets (Fig. 3A and 3D) and the four additional combinations for nested PCR approaches (Fig. 3B, 3C, 3E and 3F) to compare the DGGE patterns, anammox-specific bands and diversity of anammox bacteria detected by each set.

The results demonstrated that the primer set A438f-GC/A684r generated reproducible patterns that were unique for the different samples included in this study. DGGE fingerprints generated directly by primers A438f-GC/A684r (Fig. 3A) were similar to those from the nested PCR techniques (Fig. 3B and 3C). The DGGE fingerprints produced by both nested PCR amplifications were highly similar to each other. Seven of eight samples showed positive anammox signals from direct PCR amplification with A438f-GC/A684r (Fig. 3A). Only one sample, GS, was not amplified by direct PCR, but a nested PCR generated an anammox band for this sample. Intense DGGE bands from each sample were sequenced and included in a phylogenetic analysis (Fig. 2). All analyzed bands from primer set A438f-GC/A684r were related to anammox sequences. The BLAST results indicated them to be 97%–100% identical to uncultured anammox bacteria recovered from range of freshwater habitats. Phylogenetic analysis demonstrated that bands identified as 2-1 were associated with *Ca.* Scalindua-like sequences (Fig. 3). They were 96% and 94% identical to *Ca.* Scalindua brodae and *Ca.* Scalindua wagneri, respectively. These two identical bands, found in samples EpBr and WaBr, were at the same position as *Ca.* Scalindua control plasmid (Fig. 3A). Bands labeled as 2-2 fell into the Unknown 1 cluster, which was an anammox cluster previously reported [17]. All of the anammox bands from GW and GS were related to this Unknown 1 cluster. The majority of anammox bands found across our samples, corresponding to bands labeled 2-3, were closely related to *Ca.* Brocadia-like sequences (Fig. 2). They were 98% identical to *Ca.* Brocadia caroliniensis and *Ca.* Brocadia fulgida.

All retrieved anammox bands from the Grand River samples from nested PCR DGGE fingerprints fell into the *Ca.* Brocadia cluster. This demonstrates the potential for the direct PCR method to generate higher anammox diversity representation than that detected by either nested PCR approach for the Grand River samples (Fig. 3A, 3B and 3C). All three bands detected in WaBr (bands 2-1, 2-2, and 2-3) fell into *Ca.* Scalindua, Unknown 1 and *Ca.* Brocadia clusters, respectively, whereas all four bands from the same samples, generated by both nested PCR amplifications, were all *Ca.* Brocadia-like phylotypes. The nested PCR approach, due to probable PCR bias, underrepresented anammox bacterial diversity in our freshwater samples but can nonetheless increase amplification of anammox template from samples with low anammox bacterial abundance.

Apart from the *Ca.* Scalindua plasmid template, all PCR amplicons, with or without a nested PCR design, migrated similarly on the DGGE gel for primer set Amx368f-GC/Amx820r (Fig. 3D, 3E, 3F). In addition, only three samples, SedBr, SedBl and GW, could amplify product by direct PCR with primers Amx368f-GC/Amx820 (Fig. 3D); anammox bands from all samples can be captured by both nested PCR conditions (Fig. 3E and 3F). The DGGE patterns from this primer set were clearly different from those from the previous set, A438f-GC/A684r. All sequenced bands were indicated by the triangles. They were 99%–100% identical to the previously reported anammox-like sequences retrieved from various freshwater environments. Phylogenetic analysis revealed that anammox sequences from GW and GS still grouped together and fell into Unknown 1 cluster; all sequenced bands from the Grand River samples were affiliated with a *Ca.* Brocadia-like phylotype (Fig. 2).

All positive controls were amplified by the two primer sets A438f-GC/A684r and Amx368f-GC/Amx820, with or without nested PCR. The results were quite similar for both sets; *Ca.* Brocadia and *Ca.* Jettenia controls were close to each other on DGGE gels, but further from the *Ca.* Scalindua control. The negative control, *E. coli*, showed no signal for all primer combinations, except sets An7f/An1388r nested by both A438f-GC/A684r and Amx368f-GC/Amx820 (Fig. 3B and 3E).

In addition to the two main primer sets, A438f-GC/A684r and Amx368f-GC/Amx820r, two additional primer combinations, A438f-GC/Amx820r and Amx368f-GC/A684r were tested with and without nested PCR amplifications, following the same pattern as previously described (Fig. S1). The gradient PCR program was run at annealing temperatures between 51–60°C; the optimum temperature (sharp and bright band on agarose gel) was 55°C for both primer sets (data not shown). The primer set A438f-GC/Amx820r generated a dominant band across all samples, although Amx368f-GC/A684r produced additional DGGE bands (Fig. S1). Several representative bands from each position were sequenced and included in phylogenetic analysis (data not shown). All analyzed bands were anammox-related sequences and fell into *Ca.* Brocadia, Unknown 1 and Unknown 2 groups.

### Diversity of anammox bacteria within freshwater environments

To confirm the specificity of An7f/An1388r, A438f/A684r and Amx368f/Amx820, three representative samples were selected to construct clone libraries from each pair. Based on DGGE profiles, two sediments from the Grand River (SedBr and SedBl) and Zorra groundwater (GW) samples were included in the clone library analysis. The cloning results showed that the ratio between the numbers of colonies containing inserts and the total number of selected colonies was lower for primers An7f/An1388r (Table 3). Using this primer set, the ratio was ∼0.6 for the SedBr and SedBl libraries, but it increased to 0.9 for the GW library. The other two primer sets produced higher insert ratios, with >90% of screened colonies containing inserts (Table 3). The sequencing results revealed that only 3 out of 37 and 1 out of 36 analyzed sequences from SedBr and SedBl, constructed by primers An7f/An1388r, were anammox-related sequences, respectively. The GW library, constructed by the same primers, yielded a better result because 15 out of 25 were closely related to anammox bacteria (Table 3). The other primer sets (A438f/A684r and Amx368f/Amx820) revealed 100% specificity; all analyzed clones from all libraries were associated with anammox-related sequences. The BLAST results indicated that all clone sequences were 92%–100% identical to previously reported anammox bacteria found in various ecosystems. The number of OTUs and anammox bacterial sequences were similar for A438f/A684r and Amx368f/Amx820 libraries (Table 3). The clone sequences showing as 99% and 97% identical from each library grouped together. Representative clones from each OTU were included in phylogenetic analysis (Fig. 2). Note that phylogenetic trees based on both 1% and 3% cut-off nucleotide sequences exhibited the same anammox grouping and tree topology (data not shown). The resulting phylogeny of the clone libraries constructed by primers An7f/An1388r revealed that *Ca.* Brocadia-like sequences made up the majority of anammox bacteria found in SedBr and SedBl; GW contained anammox bacterial sequences associated with Unknown 1 and 2 clusters (Fig. 2). For primers A438f/A684r, both Grand River sediment and GW were dominated by *Ca.* Brocadia and Unknown 1 clusters, respectively (Fig. 2). Although *Ca.* Jettenia-like sequences are targeted by this primer set (Table S1), only a few clones from SedBl and GW samples fell into this cluster. Fewer clones recovered from SedBr and SedBl were closely related to Unknown 1 cluster, compared to the GW library. The expected diversity of anammox bacteria was supported by Amx368f/Amx820r libraries because the majority of anammox bacteria found in SedBr and SedBl were related to *Ca.* Brocadia-like sequences, whereas Unknown 1 cluster dominated the GW library (Fig. 2). Other minor clone library OTUs detected from GW were related to Unknown 2 and *Ca.* Brocadia clusters.

**Table 3:** Summary of the cloning results using three primer sets and three sample sites.

Primer set	Site	Total screened clone (A)	Total inserted clone (B)	B/A ratio	Anammox sequence (% recovery)	Number of OTUs		Cluster^1^
						1%	3%	
An7f/An1388r	SedBr	62	38	0.61	3/37 (8)	3	2	*Ca.* Brocadia (3/3)
	SedBl	62	39	0.63	1/36 (3)	1	1	*Ca.* Brocadia (1/1)
	GW	30	27	0.90	15/25 (60)	6	3	Unknown 1 (6/15), Unknown 2 (9/15)
A438f/A684r	SedBr	55	53	0.96	47/47 (100)	13	5	*Ca.* Brocadia (44/47), Unknown 1 (3/47)
	SedBl	70	66	0.94	61/61 (100)	11	4	*Ca.* Brocadia (56/61), *Ca.* Jettenia (3/61), Unknown 1 (2/61)
	GW	58	54	0.93	51/51 (100)	10	4	*Ca.* Jettenia (1/51), Unknown 1 (50/51)
Amx368f/Amx820r	SedBr	52	48	0.92	47/47 (100)	10	3	*Ca.* Brocadia (45/47), Unknown 1 (2/47)
	SedBl	51	48	0.94	46/46 (100)	4	2	*Ca.* Brocadia (46/46)
	GW	52	48	0.92	42/42 (100)	12	5	*Ca.* Brocadia (1/42), Unknown 1 (35/42), Unknown 2 (6/42)

1 The number of clones out of the total number of sequences affiliated with each cluster is listed in parentheses.

To test spatial and temporal changes of anammox bacterial diversity, representative sediment samples from Bridgeport (SedBr) were collected at three time points, including Summer 2010, Fall 2010 and Summer 2012. All samples were amplified by primer set A438f-GC/A684r and profiled by DGGE (Fig. S2). Overall, anammox patterns were consistent across the three time points. One additional band was apparent in the pattern from Summer 2012 and its sequence clustered with *Ca*. Brocadia phylotypes.

### Abundance of anammox bacteria in freshwater environments

**Figure 4:**
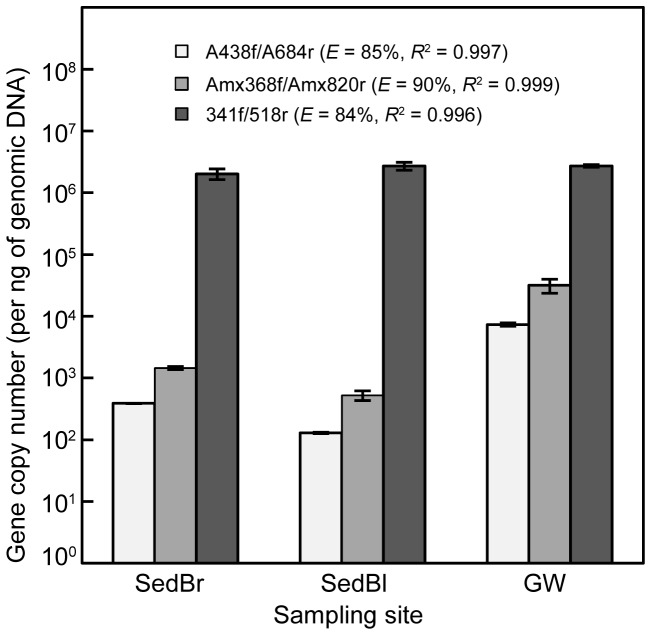
Abundance of anammox bacterial 16S rRNA genes quantified by two anammox specific primer sets, and general bacterial qPCR data for comparison. The qPCR efficiency (*E*) and coefficient of determination (*R^2^*) of each primer set are shown in parentheses.

DGGE and cloning results demonstrated that the primer sets A438f/A684r and Amx368f/Amx820 were specific for detecting anammox bacteria within freshwater environments. We used these existing two primer sets to assess the qPCR method. Total bacterial 16S rRNA gene copies were also quantified for comparison. The results showed that the measured bacterial 16S rRNA gene copies were consistent for all analyzed samples (Fig. 4). The abundance of anammox bacterial 16S rRNA genes in GW was the highest (∼10^3^–10^4^ copies per ng of genomic DNA), whereas those in SedBr and SedBl were lower and similar to each other (∼10^2^–10^3^ copies per ng of genomic DNA). The qPCR with Amx368f/Amx820r generated bacterial 16S rRNA gene abundance estimates that were approximately four times higher than for A438f/A684r in all analyzed samples (Fig. 4). Consequently, caution must be taken in using Amx368f/Amx820r to quantify anammox abundance due to possible overestimation.

## Discussion

The anammox-DGGE method developed by Moore and colleagues [17], using a nested amplification beginning with An7f/An1388r, was able to generate DGGE patterns with confirmed anammox bands for only three samples in this study (SedBr, SedBl and GW). Of 20 bands sequenced, 7 sequences were associated with anammox bacteria (Fig. 1). The remaining bands showed no relationship with any reported anammox bacterial sequences in the Genbank database. However, this method provided a reliable result for all previously analyzed groundwater samples [17], obtained from the same site as GW. Improved specificity for anammox bacterial template may be explained by a high sample-specific relative abundance of anammox bacteria, which is supported by anammox bacterial bands appearing in both bacterial and anammox-specific DGGE profiles from the GW site. Our qPCR results demonstrated that most Grand River samples contained anammox bacterial 16S rRNA genes at ≤10^2^ copies per ng of genomic DNA (data not shown), except for GW (∼10^4^ gene copies; Fig. 4). Clone library analysis was consistent with this finding because lower recovery frequencies of anammox-related clones were obtained from the SedBr (8%) and SedBl (3%) libraries, yet higher for GW (60%; Table 3). Consistent with this observation, a previous marine sediment clone library constructed by primers An7f/An1388r showed 12% anammox-related sequences [8]. Our previous groundwater libraries showed a high proportion of anammox clones with this primer set, in the range of 86%–100% [17]. Overall results suggest that primers An7f/An1388r may be specific only for anammox samples with high proportions of anammox bacteria.

To improve the detection efficiency of anammox bacteria present in low abundance, different primer combinations, with or without a nested PCR step, were tested to enhance the specificity of DGGE (Fig. 3). In this study, primer sets A438f-GC/A684r and Amx368f-GC/Amx820 were confirmed as specific for detecting anammox bacteria within freshwater environments. The A438f-GC/A684r primer pair was superior for DGGE based on well-resolved bands for both samples and controls. The resulting phylogeny revealed that direct PCR amplification by A438f-GC/A684r primers captured the most diverse anammox bacterial groups. *Ca.* Scalindua-like sequences were retrieved from picked bands only by these primers in this study. Although this cluster was normally found in marine and estuary environments and proposed to be a marine anammox-specific cluster [9], [10], [41], [53]–[56], the *Ca.* Scalindua genus has been associated with freshwater habitats such as Lake Tanganyika [35], Wintergreen Lake [22] and Lake Rassnitzer [57]. This anammox cluster was also retrieved previously from ammonium-contaminated groundwater samples [17].

Both the A438f/A684r and Amx368f/Amx820r primer sets were highly specific based on clone library analysis; all clones were affiliated with anammox bacterial sequences (Table 3). The Amx368f/Amx820r primer pair has been used for cloning in many previous studies. Reported recoveries of anammox-related clones with this primer set vary depending on the sampling sites. Clone libraries constructed from coastal marine sediment and eutrophic freshwater lake yielded 98% and 90%–100% anammox bacterial sequences, respectively [27], [36]. However, low recovery frequencies of 12%–59% were previously reported from deep-sea subsurface sediment libraries [41]. There is no prior information on the specificity of anammox bacterial community libraries generated by the primer set A438f/A684r. *In silico* analysis revealed that these four existing primers (Amx368f, Amx820r, A438f and A684r) showed high specificity and were selective for capturing known anammox *Ca.* species (Table S1). In this study, the anammox bacterial diversity recovered from cloning was in general agreement with those of the DGGE method.

The majority of anammox bacterial 16S rRNA genes found in the Grand River samples were similar to those of previously studied environments in identifying that *Ca.* Brocadia-like phylotypes were dominant in freshwater ecosystems [17], [29], [36]. The dominant anammox bacteria found in groundwater-related samples were Unknown 1 and 2 clusters. This result agreed with previous findings, showing that these unknown and uncultured groups have a potential to be a specific anammox cluster present in groundwater sites [17]. Sequences from a reductisol and ammonium contaminated aquifer also fell into a distinct group without any affiliation to known anammox clusters, being named “cluster II” in the study of Humbert and colleague (2010). These sequences were related to the Unknown 1 cluster in this study. *Ca.* Jettenia cluster was a minority population found in both the Grand River sediment and groundwater samples. The *Ca.* Jettenia lineage does not normally dominate in any specific habitat previously reported but was detectable in various terrestrial habitats [24], peat soil [39], paddy soil [18], estuarine sediments [40] and groundwater [11], [17]. Due to limited studies on the distribution of anammox bacteria in freshwater environments, future research will include more freshwater aquifers to explore their anammox bacterial communities.

The abundance of anammox bacterial 16S rRNA genes in GW was in the same range as in groundwater samples from the Zorra site previously reported [17]. In the case of SedBr and SedBl, anammox bacterial 16S rRNA genes were present in the range of 10^2^–10^3^ copies per ng of genomic DNA. River estuary sediments also contained anammox bacterial 16S rRNA in this range [23]. The qPCR results revealed that Amx368f/Amx684r captured more anammox bacterial 16S rRNA genes in all analyzed samples. Primer pair A438f/A684r could provide more accurate results than Amx368f/Amx820 because of potential false positive amplification by Amx368f/Amx820 in samples with low anammox bacterial abundance [37]. These findings are obtained mainly from the anammox bacterial 16S rRNA genes within freshwater environments; future research will include a broad range of environmental samples such as marine, terrestrial and engineered systems to evaluate the efficiency and specificity of primers for targeting anammox bacteria. Other than anammox 16S rRNA genes, functional genes such as the hydrazine oxidoreductase (*hzo*) gene have been used for anammox bacterial detection in marine sediment [8]–[10], aquatic ecosystems [11] and mangrove sediment [58]. Another functional gene marker is the nitrite reductase (*nirS*) gene, which has been used to detect anammox bacteria in the ocean [59]–[60]. The hydrazine synthase (*hzsA*) gene has also been tested as a unique biomarker for detecting anammox bacteria in both natural and built environments [61]. However, primers targeting these functional genes are still limited. Our results demonstrate that primer sets should be evaluated in a range of environments and with a careful selection of positive and negative controls to avoid false positive amplification, as seen here with An7f/An1388r. We recommend primers A438f/A684r or Amx368f/Amx820 for clone library or qPCR analyses, but only A438f-GC/A684r for DGGE-based analyses of freshwater anammox communities.

## Supporting Information

Table S1 Alignment of anammox primer sequences against known anammox Ca. species, non-anammox species of Planctomycetales and non-Planctomycetales1. The direction of all sequences is 5′-3′.
(DOCX)

Figure S1 (TIF)

Figure S2 (TIF)
