# Supplementary material for: Evaluating Primers for Profiling Anaerobic Ammonia Oxidizing Bacteria within Freshwater Environments
Source: PLoS One. 2013 Mar 7;8(3):e57242. doi: 10.1371/journal.pone.0057242 (PMC3591393; doi:10.1371/journal.pone.0057242)
Supplement: Table S1 — Alignment of anammox primer sequences against known anammox Ca. species, non-anammox species of Planctomycetales and non-Planctomycetales1. The direction of all sequences is 5′-3′. (DOCX) [file pone.0057242.s001.docx]

**­Supplemental Information**

**Evaluating Primers for Profiling Anaerobic Ammonia Oxidizing Bacteria within Freshwater Environments**

Puntipar Sonthiphand^1^ and Josh D. Neufeld^1*^

^1^Department of Biology, University of Waterloo, 200 University Ave West, Waterloo, Ontario N2L 3G1

*To whom correspondence should be addressed.

E-mail: jneufeld@uwaterloo.ca

Phone: +1 (519) 888-4567 ext. 38344

Fax: +1 (519) 746-0614

Table S1. Alignment of anammox primer sequences against known anammox *Ca*. species, non-anammox species of Planctomycetales and non-Planctomycetales^1^. The direction of all sequences is 5´-3´.

| Taxonomic affiliations  (16S rRNA gene sequences) | Genbank  Accession | An7f | An1388r | Pla46 | 1392r |
| --- | --- | --- | --- | --- | --- |
|  |  | GGCATGCAAGTCGAACGAGG | CACACCGCCCGTCAAGC | GGATTAGGCATCGAAGTC | GTACACACCGCCCGT |
|  |  |  |  |  |  |
| Known anammox *Candidatus* species |  |  |  |  |  |
| *Ca*. Brocadia sp. 40 | AM285341 | .................... | ................. | .................. | ............... |
| *Ca*. Brocadia fulgida | DQ459989 | .................... | .............---- | .................. | ............... |
| *Ca*. Brocadia anammoxidans | AF375994 | .................... | ................. | .................. | ............... |
| *Ca*. Brocadia sinica | AB565477 | .CT...T......GTT.T.A | ................. | .......AT.C.CCG..A | ............... |
| *Ca*. Brocadia caroliniensis | JF487828 | ---................. | ................. | .......AT.C.CCG..A | ............... |
| *Ca*. Scalindua sorokinii | AY257181 | .................... | .............---- | .................. | ............... |
| *Ca*. Scalindua brodae | AY254883 | .................... | ................. | .................. | ............... |
| *Ca*. Scalindua wagneri | AY254882 | .................... | ................. | .................. | ............... |
| *Ca*. Scalindua arabica | EU478624 | .................... | ................. | .................. | ............... |
| *Ca*. Scalindua sinooilfield | HM208769 | -------------------- | ----------------- | ------------------ | --------------- |
| *Ca*. Scalindua zhenghei | GQ331167 | -------------------- | ----------------- | ------------------ | --------------- |
| *Ca*. Scalindua marina clone 12C | EF602039 | ..................N. | ................. | .................. | ..N............ |
| *Ca*. Kuenenia stuttgartiensis | AF375995 | .................... | ................. | .................. | ............... |
| *Ca*. Jettenia asiatica | DQ301513 | .................... | ................. | TAC.....T.....GAGT | ............... |
| *Ca*. Anammoxoglobus propionicus | DQ317601 | .................... | ................. | .................. | ............... |
|  |  |  |  |  |  |
| Cultured non-anammox species  of Planctomycetales | | | | | |
| *Pirellula* sp. | X81942 | .......G.....TG..C.A | ................. | ...........GCG.... | ............... |
| *Pirellula staleyi* | M34126 | .............TG..C.A | ................. | ...........GC..... | ............... |
| *Rhodopirellula* sp. SM34 | FJ624371 | .............T.....A | ................. | ...........GC..... | ............... |
| *Rhodopirellula baltica* | FJ624346 | .............C.....A | ................. | ...........GC..... | ............... |
| *Blastopirellula marina* | HE861893 | .............CG...TA | ................. | ...........GC..... | ............... |
| *Blastopirellula* sp. LHWP2 | JF748733 | ..............G....A | ................. | ...........GC..... | ............... |
| *Gemmata* sp. IIL30 | JX088244 | .............GGG..AT | ................. | ...........GC..... | ............... |
| *Gemmata obscuriglobus* | X85248 | .............GGG..AT | ................. | ...........GC..... | ............... |
| *Isophaera* sp. | X81958 | .................G.C | ................. | ...........GC..... | ............... |
| *Isosphaera pallida* ATCC 43644 | NR_028892 | .................G.C | .......T......... | ...........GC..... | ............... |
| *Schlesneria paludicola* | AM162407 | ..............GG...A | ................. | ...........GC..... | ............... |
| *Singulisphaera* sp. Io4-3 | GQ889443 | -......-.........G.C | ................. | .....G..G..A.CC.CG | ............... |
| *Singulisphaera rosea* | FN391026 | .............T...G.C | ................. | ...........GC..... | ............... |
| *Planctomyces limnophilus* DSM 3776 | NR_029225 | ..............GG...A | ................. | ...........GC..... | ............... |
| *Planctomyces maris* | X62910 | -------------------- | ................. | ------------------ | ............... |
|  |  |  |  |  |  |
| Other bacteria  (non Planctomycetales) | | | | | |
| *Actinobacterium* YJF2-33 | FJ405887 | CA...............G.. | T.GCTAA.G.A.T...T | .......AT.C.CTG.TA | ............... |
| *Chloroflexi bacterium* | HQ675640 | TA.................. | A.GCAAA.G.G.T...T | .......AA.C.CCG.TA | AAGTTG......T.G |
| *Escherichia coli* | J01695 | CA...............GTA | G.GCTAA.G.G.T...T | .......AT.C.CTG.TA | ..C...G...TAAAC |

| Taxonomic affiliations  (16S rRNA gene sequences) | Genbank  Accession | Amx368f | Amx820r | A438f | A684r |
| --- | --- | --- | --- | --- | --- |
|  |  | TTCGCAATGCCCGAAAGG | GGGCACTAAGTAGAGGGGTTTT | GTCRGGAGTTADGAAATG | GAGAGTGGAACTTCTGGT |
|  |  |  |  |  |  |
| Known anammox *Candidatus* species |  |  |  |  |  |
| *Ca*. Brocadia sp. 40 | AM285341 | .................. | ...................... | .................. | .................. |
| *Ca*. Brocadia fulgida | DQ459989 | .................. | ........G............. | .................. | .................. |
| *Ca*. Brocadia anammoxidans | AF375994 | .................. | ...................... | ...............C.. | .................. |
| *Ca*. Brocadia sinica | AB565477 | .................. | ...................... | ...............T.. | .................. |
| *Ca*. Brocadia caroliniensis | JF487828 | .................. | ...................... | ...............G.. | .................. |
| *Ca*. Scalindua sorokinii | AY257181 | .................. | .................AA..A | .................A | ....A............. |
| *Ca*. Scalindua brodae | AY254883 | .................. | ..................A..A | ......G........... | ....A............. |
| *Ca*. Scalindua wagneri | AY254882 | .................. | .................AA..A | ....A.G..........A | .................. |
| *Ca*. Scalindua arabica | EU478624 | .................. | ..................A..A | .................. | ....A............. |
| *Ca*. Scalindua sinooilfield | HM208769 | ------------------ | .....................- | ------------------ | .................. |
| *Ca*. Scalindua zhenghei | GQ331167 | ------------------ | ..................---- | ------------------ | ....A............. |
| *Ca*. Scalindua marina clone 12C | EF602039 | .................. | .................AA..A | .................. | .G..A.......C..... |
| *Ca*. Kuenenia stuttgartiensis | AF375995 | .................. | ...................... | .................. | .................. |
| *Ca*. Jettenia asiatica | DQ301513 | .............G.... | .......CG............. | ...............G.. | .................. |
| *Ca*. Anammoxoglobus propionicus | DQ317601 | .............C.... | .......CG............. | ................G. | .................. |
|  |  |  |  |  |  |
| Cultured non-anammox species  of Planctomycetales | | | | | |
| *Pirellula* sp. | X81942 | .CG......GA......T | .A......GTCT.G....ACCC | TGGT....CGG....... | .TAT.C.....AGA.... |
| *Pirellula staleyi* | M34126 | .CG......GG......C | .A.....GGACC.GA.CTC.GC | TGGT....CGG....... | .TA..C......GA.... |
| *Rhodopirellula* sp. SM34 | FJ624371 | ..G......GG......C | .A.....GG..T......AC.. | TGGT....CGG....... | .T..TG.......A.... |
| *Rhodopirellula baltica* | FJ624346 | .CG......GA......T | .A......GGCT.....AAC.. | TGGT....CGG....... | .T..TG.......A.... |
| *Blastopirellula marina* | HE861893 | ..G......GG..C...C | .A.....GGA.C......AC.C | TGGT....CGG....... | .T...C......GA.... |
| *Blastopirellula* sp. LHWP2 | JF748733 | .CG......GA..C...T | .A.....GGA.C.....ACC.C | TGGT....CGG....... | .T...C......GA.... |
| *Gemmata* sp. IIL30 | JX088244 | CCGT...GC.A....... | ...T....GA...TA..C.AGA | AGGT....CGG....... | .CAT.C......GTA... |
| *Gemmata obscuriglobus* | X85248 | .CG......GGG.C..CC | ...T....GA...TA.ACCAGA | AGGT....CGG....... | .CAT........GTA... |
| *Isophaera* sp. | X81958 | .CG......GG..C...C | .A.....GG....G...C.CGC | CGGT....CGG....... | .G.TC.........C... |
| *Isosphaera pallida* ATCC 43644 | NR_028892 | .CG......GG..C...C | .......TG.C..T...T.CG. | CGGT....CGG....... | .GACTC........C... |
| *Schlesneria paludicola* | AM162407 | .CG......GA......T | .AA....GGAGGA.....GC.. | CGGT....CGG....... | .T.T..........C... |
| *Singulisphaera* sp. Io4-3 | GQ889443 | .CG......GG..C...C | ...T...GGA...G...C.CGC | CGGT....CGG....... | .GACC.........C... |
| *Singulisphaera rosea* | FN391026 | .CG......GG..C...C | .......GG....G...C.CGC | CGGT....CGG....... | .GAGC.........C... |
| *Planctomyces limnophilus* DSM 3776 | NR_029225 | .CG......GG......C | .A.....GG.GGAG..ACGGG. | CGGT....CGG....... | .T.TAC........C... |
| *Planctomyces maris* | X62910 | .CG......GA..C...T | .A.T....GT..GGA..AGC.. | CGGT....CGG....... | .T.TAC........C... |
|  |  |  |  |  |  |
| Other bacteria  (non Planctomycetales) | | | | | |
| Actinobacterium YJF2-33 | FJ405887 | G.G..CCAA....T.... | ........G..GTG....A..A | TGGT.T..CGG....... | ..TCAC....T.C..... |
| Chloroflexi bacterium | HQ675640 | .G.A.....GG......C | CTT.GGGTC...A..C.C.... | CGGT.T...GG....... | .GAG......T.C.C... |
| *Escherichia coli* | J01695 | G......GA..AA.G... | AACTTT...A.T..A.A....G | AGGT.T..CGG....... | .G.G..A...T.C.A... |

^1^Perfect matches for 16S rRNA gene primers and analyzed sequences are represented by dots (.) and gaps are represented by dashes (-). Capital letters represent mismatches with primer sequences.

Figure S1. Denaturing gradient gel electrophoresis (DGGE) profiles of anammox bacterial 16S rRNA genes. The two optional primer sets (A438f-GC/Amx820r and Amx368f-GC/A684r) were used to generate the anammox-fingerprints (A and D) by direct PCR amplification. The additional four patterns (B, C, E and F) were produced by the nested PCR assay, using primers An7f/An1388r or Pla46/1392r, followed by these two optional sets. Triangles indicate representative bands that were associated with anammox bacteria. Triangle colours correspond to the phylogeny shown in Fig 2.

Figure S2. Anammox bacterial DGGE profiles of SedBr, collected in Summer 2010, Fall 2010 and Summer 2012. All samples were directly amplified by primers A438f-GC and A684r. Triangles represent sequenced bands that were included in phylogenetic analysis. Triangle colours correspond to the phylogeny shown in Fig 2.
