# Supplementary figures and images for: Evaluating Primers for Profiling Anaerobic Ammonia Oxidizing Bacteria within Freshwater Environments
Source: PLoS One. 2013 Mar 7;8(3):e57242. doi: 10.1371/journal.pone.0057242 (PMC3591393; doi:10.1371/journal.pone.0057242)

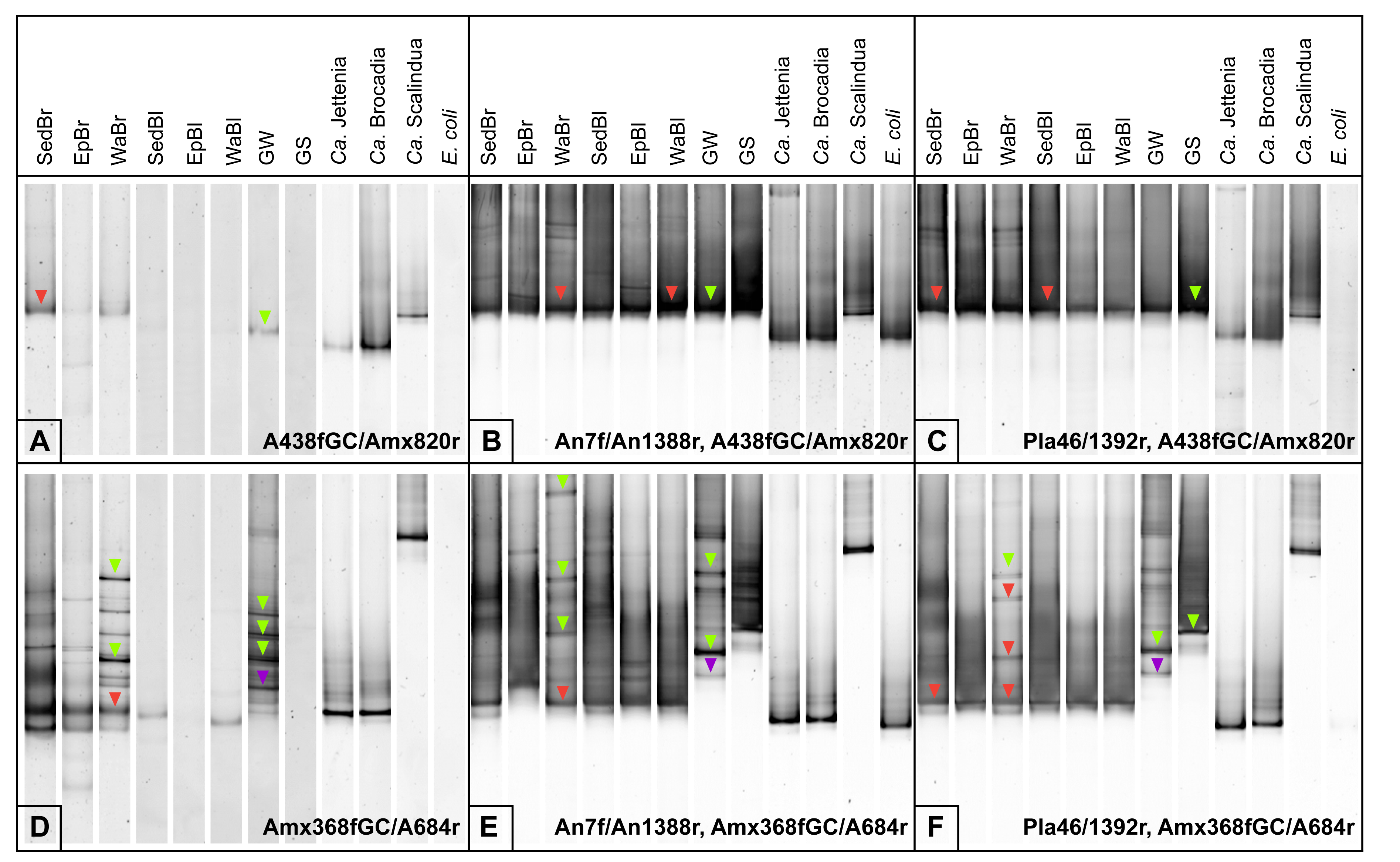

Supplement: Figure S1 — (TIF) [file pone.0057242.s002.tif]

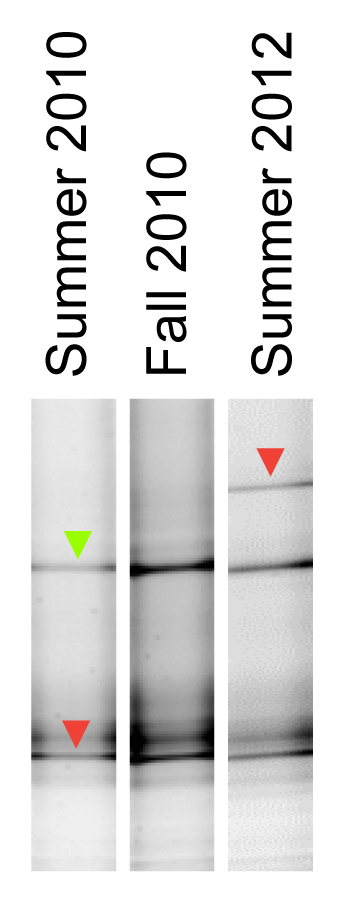

Supplement: Figure S2 — (TIF) [file pone.0057242.s003.tif]
